# Trust in telemedicine portals for rehabilitation care: an exploratory focus group study with patients and healthcare professionals

**DOI:** 10.1186/s12911-016-0250-2

**Published:** 2016-01-27

**Authors:** Lex Van Velsen, Sabine Wildevuur, Ina Flierman, Boris Van Schooten, Monique Tabak, Hermie Hermens

**Affiliations:** 1 Telemedicine group, Roessingh Research and Development, P.O. box 310, 7500AH Enschede, The Netherlands; 2 University of Twente, P.O. box 217, 7500AE Enschede, The Netherlands; 3 Waag Society, Sint Antoniesbreestraat 69, 1011HB Amsterdam, The Netherlands; 4 VU University, Talma Institute, De Boelelaan 1081, 1081HV Amsterdam, The Netherlands; 5 Roessingh Center for Rehabilitation, P.O. box 310, 7500AH Enschede, The Netherlands

**Keywords:** Telemedicine, Trust, Rehabilitation care, Portals, Activity sensors, Design

## Abstract

**Background:**

For many eServices, end-user trust is a crucial prerequisite for use. Within the context of Telemedicine, the role of trust has hardly ever been studied. In this study, we explored what determines trust in portals that facilitate rehabilitation therapy, both from the perspective of the patient and the healthcare professional.

**Methods:**

We held two focus groups with patients (total *n* = 15) and two with healthcare professionals (total *n* = 13) in which we discussed when trust matters, what makes up trust in a rehabilitation portal, what effect specific design cues have, and how much the participants trust the use of activity sensor data for informing treatment.

**Results:**

Trust in a rehabilitation portal is the sum of trust in different factors. These factors and what makes up these factors differ for patients and healthcare professionals. For example, trust in technology is made up, for patients, mostly by a perceived level of control and privacy, while for healthcare professionals, a larger and different set of issues play a role, including technical reliability and a transparent data storage policy. Healthcare professionals distrust activity sensor data for informing patient treatment, as they think that sensors are unable to record the whole range of movements that patients make (e.g., walking and ironing clothes).

**Conclusions:**

The set of factors that affect trust in a rehabilitation portal are different from the sets that have been found for other contexts, like eCommerce. Trust in telemedicine technology should be studied as a separate subject to inform the design of reliable interventions.

## Background

Trust has been studied widely within the context of technology acceptance and use, and is generally seen as an important antecedent of the acceptance and use of, and loyalty towards eServices (services provided via the Internet) [1–3]. This is also the case for telemedicine, where trust has been found to be an important antecedent of patient acceptance [4], patients’ and healthy persons’ thoughts on the usefulness of a personal health record [5], and physicians’ intention to use a telemedicine service [6]. Telemedicine refers to health services that enable patients to receive treatment in their daily living environment, whereby distance is bridged by ICT, and at least one healthcare professional is involved [7]. Models that explain trust have been developed for online services within contexts like eCommerce [8] or eGovernment [9], but are lacking for telemedicine. And telemedicine is a different kind of service: End-users (patients) do not purchase a good or service, but use a technology as part of treatment, while the party offering the service is a medical organization that bought the technology from a developer and offers it free of charge (depending on the nature of the technology and the applicable healthcare insurance or government regulations) [10].

Trust in online services has been defined in many different ways. Some authors see trust as one-dimensional. For example, Corritore and colleagues defined this type of trust as “an attitude of confident expectation in an online situation of risk that one’s vulnerabilities will not be exploited” [11]. Other authors have posited that trust is two-dimensional (whereby trust in the organization providing the technology and trust in the technology itself make up trust in an online service [12]), or even multi-dimensional. The most well-known models for trust in online services have applied a multidimensional approach. Mcknight et al. [8] have developed a very elaborate model of trust in web vendors whereby trust is affected by an individual’s disposition to trust, experience with the Internet as a trustworthy medium, perceived website quality, and trust in the organization behind the website; Gefen et al. [13] came to a model of trust in online shopping in which trust is affected by trust in the organization providing the service, situation normality (i.e., a situation in which a person’s expectations of how a certain service should go about are confirmed) and structural assurances (e.g., guarantees, seals of approval). Within the context of this article, we see trust as a reliance on a health organization by its end-users and stakeholders with regard to the service it provides (and the factors that make up this service) in both an online and offline setting. By defining trust this way, we focus the current work on the formation of trusting beliefs: perceptions of specific attributes of the provider of the eService [8]. Positive trusting beliefs are a prerequisite for positive trusting intentions. Positive trusting intentions, on their turn, are a prerequisite for trust-related behavior (such as using a service). This line of thought assumes that people go through several trust-related steps before actual use of a service and that each of these steps must have a positive outcome before the final, desired behavior takes place. Such reasoning can also be found in similar behavioral models such as the theory of planned behavior [14] and the technology acceptance model [15].

A lot of research has focused on identifying what makes up consumer trust in online health information when searching the Internet for general information about a condition or for informing oneself before making a medical decision [16, 17]. In these cases, factors like source credibility and the currency of information (i.e., how up-to-date information is) play an important role. Telemedicine is part of patient treatment and it can be expected that trust works differently here, as it is likely to be influenced by relationships with a healthcare professional or a healthcare organization. However, the research on trust in telemedicine services is still in a stage of infancy [18], and only a few studies have made a first attempt at identifying factors that affect trust in telemedicine services. Physicians’ trust in electronic health care records was found to be influenced by perceived risk and information integrity [19] and healthcare professionals’ trust in an adverse event reporting system was found to be influenced by the subjective norm [6]. Brown and colleagues [20], finally, stated that the disposition to trust any telemedicine service by healthcare professionals is dependent on personality traits (e.g., one’s need for control or competitiveness). The aforementioned studies show that trust in telemedicine services will probably be multidimensional. However, the factors that have been identified so far to explain trust in telemedicine services do not disclose a coherent picture and are only approached from the healthcare professionals’ perspective.

In this study, we took an empirical, exploratory approach to map a specific instance of trust, namely trust in a telemedicine service. As such, the current study draws from the broader literature on trust in medical services [e.g., 21, 22] and contributes to the body of knowledge on trust and eService design for the healthcare domain. More specifically, we mapped the concept of trust in relation to a rehabilitation portal and determined what makes end-users (dis)trust this rehabilitation portal. As we focus on trusting beliefs, our aim was to identify the factors or issues that affect people’s perception of this rehabilitation portal’s trustworthiness. Whenever we talk of ‘trust’ in the remainder of the article, we are talking about trusting beliefs, not the actual act of trusting someone or something (trusting behavior). The portal under investigation is used within the treatment of individual patients (e.g., Chronic Obstructive Pulmonary Disease, chronic pain), includes many features (provision of disease-related information, an electronic patient file, remote exercising, and the use of sensor technology), and therefore makes an interesting case study for assessing trust in state-of-the-art rehabilitation portals. The results of this study can serve as a first basis for developing a fine-grained understanding of what makes up trust in telemedicine portals for rehabilitation care (both from a patient’s and a healthcare professional’s view). Such information is crucial for designing these services and implementation strategies that take end-users’ concerns about trust into account and thus, have a higher chance of success.

## Methods

We investigated the concept of trust in rehabilitation portals by means of semi-structured focus groups with patients and healthcare professionals. Focus groups were chosen as a method as they are a good means to explore a new concept [23].

### Study context

All participants were recruited via two large rehabilitation centers in the Netherlands to which the researchers had access: One in the metropolitan region of Amsterdam, and one in the rural region of Enschede. This resulted in two focus groups with patients and two focus groups with healthcare professionals. We split these two groups to allow the patients to speak freely about their treatment and care providers (otherwise it was possible that patients would end up with members of their care team in the same focus group). Healthcare professionals were recruited by the researchers, while patients were recruited by the researchers (in Enschede) or by healthcare professionals (in Amsterdam). We aimed for a group size of 4 to 8 participants, whereby we recruited more people to account for persons who declined at a late moment [24].

During the focus groups, we made use of screenshots of a portal of a large, Dutch rehabilitation center. This portal is provided to patients as soon as they start their treatment in the rehabilitation center and allows them to manage appointments, complete questionnaires, or exercise at home by using a personal schedule and online instruction videos. This allows patients to exercise at home, without the aid of professionals. Professionals can use the portal to monitor patients and for providing personalized rehabilitation programs. Next, the use of activity sensors for informing treatment was demonstrated by explaining how activity data can be recorded by means of a hip-worn sensor and how this data can be displayed to the patient and the healthcare professional. The professional can then make use of this data during a consultation for advising patients on how to distribute their energy over the day in a well-balanced manner. For more information on the development and workings of the rehabilitation portal, see [25]. We selected this portal as a discussion starter, as it entails different functionalities that utilize different kinds of (personal) data, including activity monitoring (which can be considered to be a ‘quantified self’ application). Therefore, we expected it to evoke a lot of discussion about end-users’ thoughts on the value of technology (as part of treatment), and the storage and use of personal data.

### Focus group setup

Each focus group started with an introduction of its goal. Then, the participants provided informed consent and gave permission for audio recording. The participants were assured that they would remain anonymous and that their decision to participate would not affect their treatment or their professional position in any way. Due to the nature of the study (an exploratory study with adult volunteers that aimed to inform information systems design), approval from an ethics committee was not necessary [26]. The study was officially exempt from medical ethical assessment by the Medical Ethical Committee of Twente. We anticipated that it would be difficult for participants to verbalize their thoughts about a concept as abstract as trust. To help them, we used visual materials to support all parts of the session, such as large sheets of paper on which the participants could draw and write down their thoughts.

**Fig. 1:**
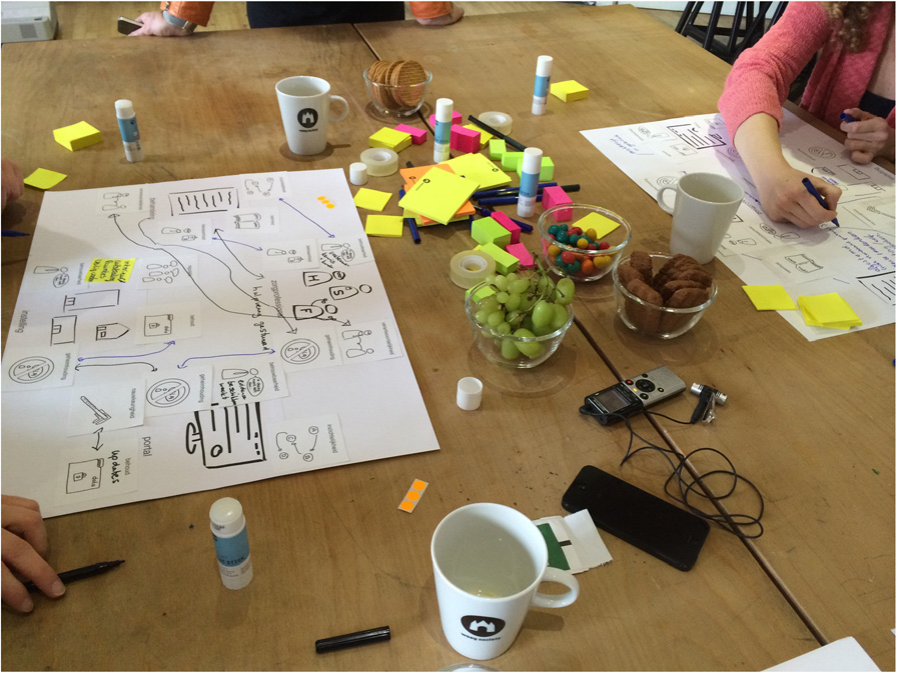
Healthcare professionals creating models of trust
- *Trust cues*. The patients were shown screenshots of a rehabilitation portal in which cues were embedded that have been found to increase trust in online services: Login procedures with different authentication technologies [28], disclaimers [29], and the visual appearance of the homepage [30]. Then they were asked what they thought about these cues. Do they in- or decrease trust? And why? The professionals were shown screenshots of the professionals’ side of the portal and were asked to comment on the interface and interaction design of 1) the homepage, 2) the patient overview, and 3) a screen depicting the current status of a patient. We opted for visual design as the primary subject of discussion as the login procedure and disclaimer do not play a prominent role in this part of the portal, and because visual design and navigation design play a crucial role in forming trust [31].
- *Sensor data.* All participants were questioned about using activity sensors to inform patient treatment, and asked to make a distinction among the stakeholders that they think can be trusted to store or use this kind of data. The latter was done by letting the participants place cue cards with different stakeholders (e.g., general practitioners, insurance companies) on a sheet of paper that depicted a continuum (ranging from ‘not allowed’ to ‘allowed’). Finally, they were asked to state a preference for a large-size activity sensor (the ProMove2, 65 × 50 × 30 mm, 70 g; to be worn with a belt-clip) and data storage by a care organization, or a small activity sensor and data storage by a commercial organization (the Fitbit one, 48 × 19 × 10 mm, 8 g; can be worn in a pocket or clipped to a belt or bra). This was done to trigger a discussion between sensor usability and privacy.

The focus groups consisted of four parts:

- *Patient journeys.* Individually, patients constructed their patient journey (an outline of the process that an individual patient follows from the patient’s perspective [27]) on a large sheet of paper and indicated where trust played an important role. The professionals were shown a typical patient journey that included the use of sensor technology and web-based training. Each professional marked down, individually, where they thought trust between patient and healthcare professional, and/or human and technology plays an important role.
- *Models of trust.* Both, patients and healthcare professionals noted down what, for them, makes up trust in four factors which we anticipated would affect trust in a telemedicine service, offered by a rehabilitation center: Healthcare professionals, the rehabilitation center, the treatment, and the rehabilitation portal. They did so in pairs to stimulate discussion among each other. Participants could stick cue cards to a large sheet of paper on which the four subjects were displayed. These cue cards included the following terms: Reputation, competence, conservation, accuracy, responsibility, secrecy, transparency, controllability, authenticity, openness, reliability, and safety. However, participants were also allowed to add self-devised terms. See Fig. 1 for an example.

### Data analysis

During the focus groups, all participants made their own visualizations of patient journeys and models of trust. Two analysts (LvV and IF) grouped similar responses (separately for patients and healthcare professionals) to identify which factors were named most often when stating what makes up trust in a healthcare professional, rehabilitation center, treatment, or portal technology. Any disputes were resolved by discussion. Next, the audio recordings were analyzed on a per-question basis, using inductive thematic analysis [32]. For each predefined question that was posed, similar answers were grouped and we determined whether there was no agreement among the participants, or whether some, or half, or (almost) all participants gave the same answer.

## Results

First, we discuss the results of the focus groups with patients. Then, we report the results of the sessions with healthcare professionals. For each participant group, we discuss the results related to the patient journeys, trust models, trust cues, and sensor data.

### Patients

We held one focus group with nine patients in Enschede (five women and four men; ages ranging from 45 to 60 years; suffering from conditions like cerebrovascular accidents and chronic back pain) and one in Amsterdam with six patients (four women and two men; ages ranging from 41 to 66 years; suffering from conditions like chronic pain and a cerebral infarction). None of the patients that participated had experience with the telemedicine technology that was presented during the focus groups.

The journeys that the patient participants experienced during their care were all unique, but there were several commonalities among the situations in which they thought trust was important:

- ■ When a patient was referred from one healthcare professional to the next. Patient participants expected the professional to whom they were referred, to be very trustworthy;
- ■ When diagnostics was performed. This increased trust as the patient participants then believed that his or her problems were dealt with;
- ■ When patient participants thought their situation was taken seriously. This increased trust in the healthcare professional;
- ■ When a patient participant felt very dependent on the healthcare professional. This increased trust out of necessity;
- ■ During the first face-to-face meeting between a healthcare professional and a patient. This is the moment where, the patient participants stated, the decision was made for a patient to trust his/her healthcare professional or not.

**Fig. 2:**
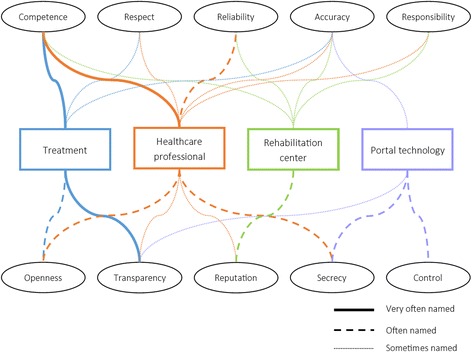
Terms that patient participants associated with trust in treatment, a healthcare professional, a rehabilitation center, and portal technology

In pairs, the patient participants created drawings that depicted what they thought makes up trust in a treatment, healthcare professional, rehabilitation center and portal technology. Terms that they noted down, but that were not provided on the cue cards were: Participation, seriousness, insight in data, result-driven, technological issues, accessibility, attention, and involvement. See Fig. 2 for the general trends in the overviews. Terms that were only named once or twice are not included.

For a treatment to be trusted it must be good (competence) and it must be clear what it entails (transparency). Many things determined trust in a healthcare professional, but most importantly his/her competence, while trust in the rehabilitation center was mostly derived from its reputation. Portal technology, finally, was trusted when the patient participants perceived they were in control, and their data was kept private.

#### Logging in

When confronted with the log in screen of the rehabilitation portal, the patient participants named several things that increase their trust when logging in at such a service:

- ■ The presence of ‘HTTPS’ in the browser address bar (which denotes the use of a secure connection, instead of the regular, unsecure connection that starts with ‘HTTP’). The patient participants indicated they have learned to watch for the s in HTTPS as a result of large-scale media campaigns by banks.
- ■ The presence of a familiar logo. The patient participants indicated they looked for the logo of the rehabilitation center they were being treated at.
- ■ The right way of providing and managing login data. A considerable number of patient participants indicated that they disliked it when they receive a set of automatically generated login data from an organization. Rather, they would like to login with their email address as an identifier, after which they can choose a password themselves.

The use of the national authentication technique for Dutch eGovernment websites (DigiD) appeared to diminish trust in the portal. Due to negative media coverage about DigiD, many patient participants were wary about the mechanism’s safety. A few patient participants, however, liked DigiD as it was familiar to them and required them to remember only one set of login data.

#### Disclaimer

Almost all patient participants were negative about disclaimers and stated that they actually decreased trust in portal technology. They believed that the presence of a disclaimer is a way for a care organization to get rid of its responsibility and is used as an excuse for providing bad technology without having to deal with the consequences. As two patient participants discussed:

“That ‘they are not responsible’ [in the disclaimer text], that’s some kind of thing… Like, ‘sorry that things went wrong, but it’s not our fault.’”

“Yes, they cover up for themselves. That does give you a kind of feeling.”

“Like, we didn’t think this through very well, so let’s put in a disclaimer.”

#### Homepage

When looking at the homepage of the rehabilitation portal, several patient participants mentioned two aspects that decreased their trust. First, the homepage did not appear personal. The patient participants mentioned that they did not think it was their personal space, as their name was not mentioned anywhere. Second, several patient participants stated that they perceived a lack of control over their personal data: They wanted to be able to change things themselves (such as their name, address, etc.).

After demonstrating the large and small activity sensor and the purposes they can serve for rehabilitation care, the patient participants gave mixed reactions. Some of them preferred the small sensor, as they liked the usability, and had no problems with commercial storage of this kind of data. As two of them discussed:

“It doesn’t matter to me. When I’m carrying stuff all day and someone reads that. Well…”

“When you’re very active in the morning, but not in the afternoon. That’s of no use to anybody.”

Other patient participants valued private data storage and therefore preferred the large activity sensor whereby data could be stored on the servers of a rehabilitation center. Finally, we asked the patient participants to indicate, individually, who they thought should be permitted to store activity data, and who should be permitted to view activity data. Patient participants were fine with rehabilitation centers, their general practitioner, hospitals and researchers storing their data. Those that were allowed to view the data were mostly medical specialists, physical therapists, their general practitioner, and researchers. The group of stakeholders that was not allowed to store data was similar to the group that was not allowed to view data. They were, predominantly, healthcare insurance companies, commercial companies, and health and safety officers.

### Healthcare professionals

Again, we held two focus groups. One was held in Enschede with nine healthcare professionals (three women and six men; ages ranging from 35 to 63 years; professions included physical therapists, psychologists, and a speech and language therapist) and one was held in Amsterdam (four women; ages ranging from 25 to 35 years; professions were occupational therapist, psychologist, or physical therapist). None of the professionals that participated had experience with the specific telemedicine technology that was presented during the focus groups. However, all professionals had worked with telemedicine technology in the course of their work (including rehabilitation portals and activity sensors for informing treatment).

The participating professionals’ thoughts on when trust mattered most to them during a patient journey focused on a few moments:

- ■ During the first and last face-to-face meeting between patient and healthcare professional. Especially as during the first meeting the initial trust between the two parties was set.
- ■ During the creation of the electronic patient file. A new patient’s file should be correct and complete, and should be stored securely. Without these guarantees, the participating professionals felt they could not trust the use of this data.
- ■ When using sensor data to inform patient treatment. The participating professionals indicated that they needed to be sure that sensor data is reliable.
- ■ When communicating with a patient via an online portal, professionals stated, it should be guaranteed that this communication cannot be read by an outsider.

**Fig. 3:**
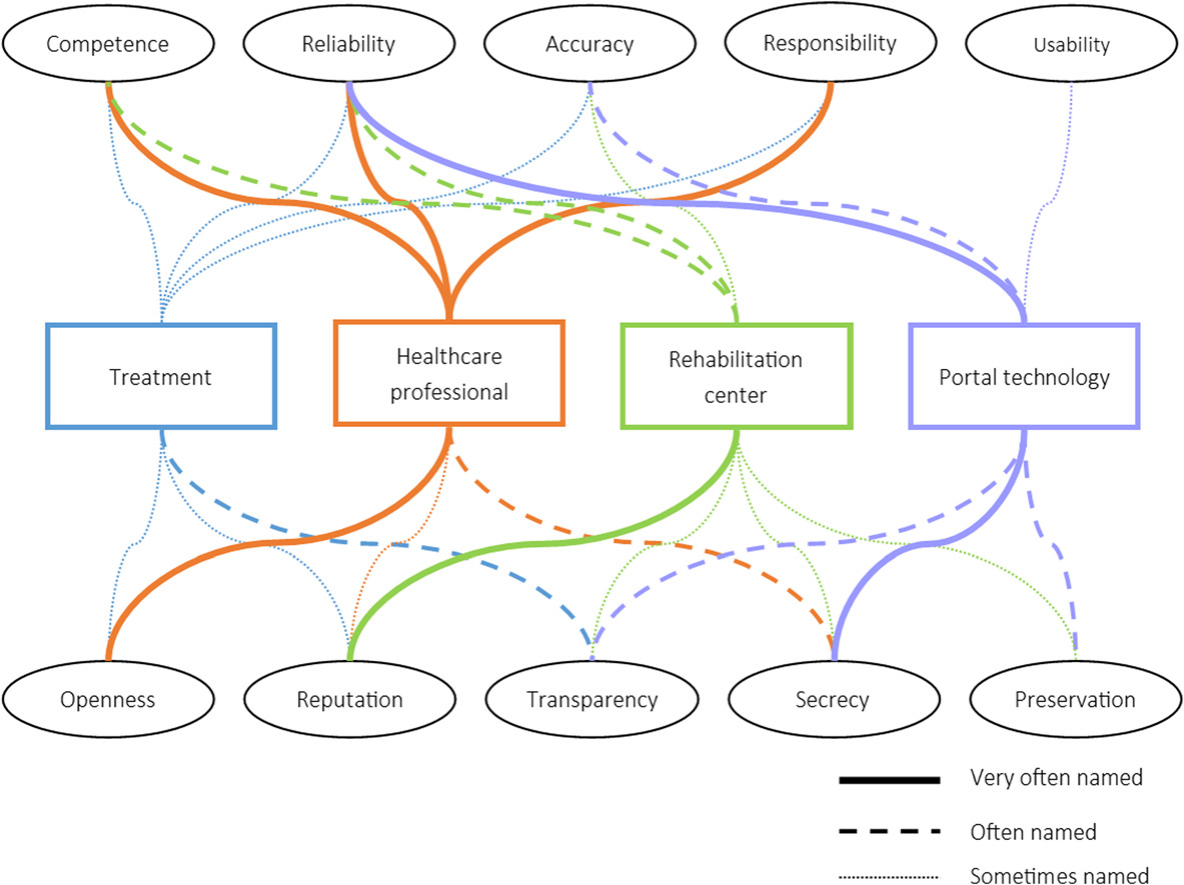
Terms that participating professionals associated with trust in treatment, a healthcare professional, a rehabilitation center, and portal technology

Figure 3 displays the terms that the participating professionals associated with trust in the different concepts. Terms that participants noted down, but that were not provided on the cue cards were: Honesty, professionalism, usability, ownership of data, tangible aspects, demand-driven, collaborative, and personal.

Trust in the treatment was made up of a myriad of factors, none of which appeared to be the single most important one. Trust in a healthcare professional appeared to be made up of his/her reliability, competence and sense of responsibility mostly. Reliability of the healthcare professional appeared to be an umbrella term, but mostly denoted a good understanding between the healthcare professional and the patient. Trust in the rehabilitation center was mostly the result of its reputation. Trust in rehabilitation portal technology, finally, was mainly determined by its reliability and the degree to which privacy is possible. When we asked the participants what they understood when talking about the reliability of portal technology, one participant summarized this as a combination of technical reliability and scientifically proven effectiveness:

“That it works properly; is not constantly offline. But also scientifically reliable.”

#### Homepage

When we showed the participating professionals the homepage of the rehabilitation portal, many commented on its clarity and ease of use. They also stated, however, that this is not related to trust in the technology. Trust, they stated, is mainly affected by the policies behind the portal.

“It looks very clear to me, but to me that’s independent of trust. Because, what happens with the data that is inserted? And I don’t have to see this from a screen, that’s just [policy].”

A difference in the participating professionals’ disposition to trust could be seen between the two focus groups. In Enschede, many had negative experiences with telemedicine technology as provided by their employer. In Amsterdam, they were relatively inexperienced. Therefore, in Enschede half of the participating professionals distrusted the technology:

“I don’t think it will be any good. I have very little trust in my managers, my department. At this organization, people put very little thought in lots of things. I have a lot of distrust regarding my employer.”

The other half assumed that a technology provided by their employer would be safe:

“When I get a letter from my employer, stating, “We are going to work with that system”, then I assume it’s reliable. Because they know about privacy and protection of patient data, and then I assume everything has been taken care of. And that that environment is completely safe and secure.”

In Amsterdam, the participating professionals were more trusting of telemedicine technology in general and named several aspects that increased their trust in the rehabilitation portal:

- ■ The presence of a secure connection, as denoted by HTTPS in the address bar;
- ■ The presence of a logo of a reliable care organization;
- ■ The clarity of the screen;
- ■ The fact that a healthcare professional must log in, after which his or her name appears on the screen. To the participants, this indicated that an individual is responsible for his or her actions on the portal, and could be held accountable.

One question that the participating professionals in Amsterdam had was whether or not the system would automatically log out a user after a period of inactivity. This would increase their trust in the portal, as misuse would be prevented this way.

#### Patient overview

When we showed the page displaying a professional’s patients, we received several comments on usability flaws that decreased the participating professionals’ trust in the data (the flaws were related to missing birth dates and contextual information on the overview page).

#### Patient status

As on the previously shown pages, the participating professionals missed contextual information on the screen that informed them about the current status of an individual patient (including the results of an self-management diary for COPD patients). This lack of contextual information made them distrust the patient data. Next, some participating professionals stated that they found it hard to estimate the severity of a situation, based on a subjective indication of symptoms, and that they distrusted patients to truthfully answer the questions in the self-management diary:

“By default, you should not trust the patient to honestly insert his or her data. You never know exactly: They are illiterate, they give socially desirable answers. They can gain something by filling it out in a certain way.”

The healthcare professionals were given the same presentation of the activity sensor as the patients. Almost all participating professionals had trouble with trusting the data. They missed the contextual information that is necessary to interpret a patient’s activity, or did not trust a sensor to assess different kinds of activity:

“Within our group of lung patients […] there are people that are ironing clothes, and for them that’s quite intensive, but that’s something it doesn’t measure. So: does it measure what you want?”

Another participant, however, agreed with the limitations but did not see a problem, considering the goal of the sensor:

“I use it. I look at how much people move and whether or not it can then be an inhibiting factor. That’s on a very high level, and then it’s a good indication.”

Finally, some participants were afraid of patients cheating:

“I have also participated in a step counter study once, and there are quite some possibilities to influence results. You can, of course, give it to your partner. But I don’t think my patients will do that. But I do make an estimation about a patient at the start, whether or not that patient will do it correctly and honestly.”

Next, we showed the Fitbit to the participating professionals. As was the case for the patient participants, some professionals were against data storage by a commercial organization. Whereas others, again, did not see any harm. These participating professionals did draw the line, however, when commercial stakeholders would contact the patients about their activity patterns. Last, we asked the participating professionals to indicate who they thought should be allowed to view and store activity data. They indicated that only medical professionals should be allowed to store and view data, while researchers should also have the option to view it. Healthcare insurers and commercial stakeholders should, according to them, preferably not be allowed to store and view such data.

## Discussion

### Antecedents of trust in a telemedicine portal for rehabilitation care

Based on the results of this study, we listed the factors that make up trust in a rehabilitation center, a healthcare professional, a patient, a treatment, and portal technology (see Table 1). Combined, these factors shape patients’ or healthcare professionals’ trust in a rehabilitation portal, or a technology-supported health service. Included in this table are the factors that were mentioned most often during the focus groups (please note that we changed some terms that the patient participants brought forth and that are displayed in Fig. 2 to align better with the concept they are describing (e.g., ‘competence’ of the treatment was reworded into ‘effectiveness’)).

**Table 1:** Factors that make up trust in a telemedicine portal for rehabilitation care

	Factors that shape patients’ trust	Factors that shape healthcare professionals’ trust
Rehabilitation center	- Reputation - Taking responsibility for actions - Accuracy in execution of daily tasks	- Reputation - Taking responsibility for actions - Competence
Healthcare professionals	- Competence - Openness in communication - Keeps patient data secret	Not applicable
Patients	Not applicable	- Honesty - Skills - Ability to estimate patient functioning via portal
Treatment	- Effectiveness - Clarity - Collaborative decision between healthcare professional and patient	- Effectiveness - Clarity - Collaborative decision between healthcare professional and patient
Technology	- Level of control - Privacy - Data preservation - Usability	- Technical reliability - Secure data storage - Transparent policies - Usability

Table 1 shows that patients and healthcare professionals more or less agree on the factors that make up trust in the rehabilitation center that provides the portal, and the factors that make up trust in a treatment. Trust in the healthcare professional and patients are, of course, unique for each end-user group. Trust in the technology, finally, means something different for patients than for healthcare professionals, suggesting that their needs and wishes on this topic need to be addressed separately during the design of trustworthy portal technology for rehabilitation care. Several of the factors we found in this study were also identified in previous research. The competence and openness of the healthcare professional were also identified by others as important for creating trust in a physician [33], while usability has also been identified as a pivotal part of trust in eServices before [34]. We also found an indication of prior experience with telemedicine playing a role in the formation of trust beliefs among healthcare professionals, where bad experiences led to low trust. This hypothesis should be investigated further in future research.

The factors that, we found, affect trust in a telemedicine portal for rehabilitation care appeared to both overlap and differ, when compared to the factors that make up leading models that explain trust in eServices. The model by McKnight et al. [8], for example, also includes factors like perceived site quality and institution-based trust. The make-up of these factors, however, appears to be different for a medical context. The model for developing initial trust in an online company by Koufaris and Hampton-Sosa [35] identifies organization reputation, perceived ease of use, and perceived security control as important antecedents, but does not include healthcare-specific factors. We have identified several antecedents of trust that are unique for telemedicine: Trust in healthcare professionals and trust in treatment, which were identified from existing research and confirmed by participants; and trust in patients, which emerged as an important antecedent of trust for participating professionals. These factors are also not listed in overviews of factors that affect trust in online health information, where usability and rich, unbiased information play a crucial role [30]. This study suggests that when designing trustworthy telemedicine portals for rehabilitation care, we cannot rely solely on the existing models that inform us how to design trustworthy eServices or reliable online health information. Rather, future research should develop a complete, validated set of design heuristics for this context.

### Design guidelines for a trustworthy telemedicine portal for rehabilitation care

First meetings between a patient and healthcare professional should be done in a face-to-face setting, as this is the moment where the patient largely determines his or her trust in the healthcare professional. For healthcare professionals, this meeting also establishes the trust in portal technology. It is during this meeting that they first see a patients’ electronic dossier and have to determine whether it is correct and complete. While this advice may seem obvious, we are also witnessing the rise of fully self-help Internet health interventions [e.g., 36, 37]. It is important to remind ourselves that the loss of a first face-to-face meeting in such interventions may have a detrimental effect on patient trust.

Patients look for several cues to determine their trust in a rehabilitation portal: A secure Internet connection, a familiar logo (of the healthcare organization providing the technology), the option to alter login credentials and personal data, and a personal approach (e.g., a homepage with the patient’s name). The importance of these cues for building online trust has already been identified for other types of websites [38], and we have found that these cues also play a role for rehabilitation portals. Professionals also focus on several trust-increasing cues. Specifically, they focus on the presence of their name on the homepage (which ensures a sense of personal responsibility), and automatically being logged out after a period of inactivity. Next, they want the guarantee that patient data cannot be read by an outsider, for example by informing them about the policy on this point. Ideally, this should be done by complying with international standards, such as ISO [39] or EN [40], and by informing professionals of this.

Two related topics that came up several times during the focus groups were data access and control. Among both patients and healthcare professionals there appears to be a group of people that desires the possibility to select those organizations that are allowed to access patient data. We think this should be seen as a call for developers of telemedicine portals to provide patients with fine-grained mechanisms for controlling which party is allowed to view, store and/or use different types of patient data (health data, sensor data, etc.).

Some trust cues may work counter-effectively. Official, national authentication mechanisms can decrease trust, due to negative media publicity, as was the case for DigiD, the Dutch, national authentication mechanism. Disclaimers, which other researchers have found to increase trust [29], were seen by the participants in our focus groups as a bad excuse for bad design.

### Using sensor and patient-generated data to inform treatment in rehabilitation care

Healthcare professionals were highly concerned about the quality of sensor data. They thought activity sensors were inferior devices, as single sensors could not assess different kinds of activities (e.g., walking and ironing) and lacked the option to record contextual information. As a result, many of them did not want to use them for informing patient treatment. This would explain why we have witnessed professionals’ reluctance to use such sensors in the past [41]. The versatility of activity sensor data should be improved before it will be trusted and used by healthcare professionals in rehabilitation care. Special attention should be paid to including contextual information and effective sensing of a wide range of human activities.

Finally, it was an interesting result for us that healthcare professionals do not always trust the information that patients provide. They think that some patients lack the skills needed to give good information or are not honest to them. Furthermore, they also do not trust the data provided by sensors worn by patients. It is therefore important that we devise a way of communicating trustworthiness of patient-generated data for healthcare professionals.

## Limitations

The setup of this focus group study was geared towards eliciting the different factors and design considerations for creating end-user trust in rehabilitation portals. We took an explorative approach as only little work has been done on the topic so far. Our results should therefore be seen as a starting point for determining, statistically, which factors exactly make up trust in such portals, and the importance of each factor. The same holds for the creation of guidelines for designing for trust in rehabilitation portals. Future research should acknowledge and expand these findings.

We have focused, in this study, on what makes up ‘trusting beliefs’ (perceptions of specific attributes of an eService provider [8]) about a rehabilitation portal. Trusting beliefs are a first step in actually performing the desired trusting behavior, and things might go wrong along the way (e.g., a patient may have a favorable opinion of a rehabilitation portal and might have the intention to trust it as well, but loses this trust during a complicated registration procedure after which the patient decides not to trust and use the portal after all). Mapping the full life-cycle of trust formation and behavior is important to fully understand the role of trust. This, however, was outside the scope of this study and a longitudinal, observational study would be far more suitable for this goal than conducting focus groups. However, this work has laid the foundations for such a study.

The context in which this study has taken place is rehabilitation care. Results from this study may, therefore, not hold for other care contexts (such as primary care or mental care). The participating patients sometimes suffered from ‘misunderstood complaints’. Their condition was difficult to diagnose and therefore these patients have quite some history in healthcare, which may have affected their trust in healthcare professionals or care institutes. Other professions, such as general practitioners or dentists may have other views when it comes to trusting their patients and the technology they use during their work. Future research should determine to what degree the results we found are similar for other care contexts.

Finally, the example that was used as a conversation starter during the focus groups was a specific instance of a portal for rehabilitation care (that includes medical information, an electronic patient file, remote exercising, and the use of sensor technology). The collection of features that is unique for this portal may have influenced results and we should be cautious with generalizing results to other health or rehabilitation portals with a distinctly different set of functionalities.

## Conclusions

In this study, we have explored the coming about of patient and healthcare professionals’ trust in a telemedicine portal for rehabilitation care. The resulting factors that affect trust are quite different from the factors that influence trust in other domains, like eCommerce [8, 42] or eGovernment [2]. This implies that the concept of trust is distinctively different for healthcare and telemedicine portals than for other contexts, and should be dealt with in a specific manner in research and portal design. Different factors should be taken into account (e.g., trust in care organizations) and design should incorporate a set of design cues that are proven to be beneficial for trust in a rehabilitation portal (the list we present in this article can serve as a first start). Our next step is to take the models of trust that we have presented, to translate them into questionnaires, and to test and refine them on a large scale with patients and healthcare professionals. This way, we will also be able to say which factors have the most importance.

Finally, patients and healthcare professionals voiced different preferences regarding different actors and organizations storing and viewing their data and we found that providing them with control over these rights would increase trust. This strengthens the call to telemedicine developers to develop privacy-enhancing technology that allows end-users to control their data in a usable manner [43].
